# The N-terminal domain of *Chlamydia psittaci* Pmp19G modulates macrophage autophagy by targeting the NOD1 receptor and the ATG16L1–RAB7 signaling pathway

**DOI:** 10.3389/fimmu.2025.1645250

**Published:** 2025-09-10

**Authors:** Qiang Li, Huimin Wang, Yuehui Cui, Zongyang Huang, Xuedi Zhang, Rongze He, Cheng He

**Affiliations:** National Key Lab of Veterinary Public Health Security, College of Veterinary Medicine, China Agricultural University, Beijing, China

**Keywords:** *Chlamydia psittaci*, Pmp19G, NOD1, macrophages, autophagy, immune evasion

## Abstract

**Introduction:**

*Chlamydia psittaci* (*C. psittaci*), a zoonotic intracellular Gram-negative bacterium, is responsible for human infections presenting as flu-like fever and community-acquired pneumonia. Previous studies have implicated polymorphic membrane (Pmp) G in tissue tropism and induction of immune responses. However, the mechanisms by which Pmp19G promotes *C. psittaci* infection and immune evasion—especially via macrophage subversion remain poorly understood.

**Methods and Results:**

This study demonstrates that both *C. psittaci* and recombinant *C. psittaci*-specific Pmp19G protein activated autophagy in macrophages. This activation was characterized by increased autophagosome formation, conversion of LC3-I to LC3-II, and accumulation of p62/SQSTM1, while lysosomal associated membrane protein 1 (LAMP1), a late autophagy biomarker, remained unaffected. Utilizing pull-down assays coupled with co-immunoprecipitation, we identified the NOD1 receptor as an interactor with the N-terminal domain of Pmp19G. Subsequent analysis confirmed activation of the NOD1-ATG16L1 signaling pathway. NOD1 knockout or knockdown significantly impaired Pmp19G-mediated autophagic flux. Furthermore, treatment with Pmp19G enhanced the recruitment of RAB7 during the late stages of autophagy.

**Discussion:**

Our findings indicate that Pmp19G regulates macrophage autophagy through distinct mechanisms in early and late phases: activation of the NOD1-ATG16L1 signaling pathway initiates early autophagy, while enhanced RAB7 recruitment inhibits autophagosome-lysosome fusion during late autophagy. Collectively, Pmp19G-involved manipulation of the autophagic process represents a critical strategy employed by *C. psittaci* to evade host immune defenses, leading bacterial survival and spread.

## Introduction

*Chlamydia psittaci* (*C. psittaci*) is an obligatory intracellular pathogen that parasitizes within eukaryotic cells. It poses a significant zoonotic threat, infecting poultry, livestock, wild animals, and humans, leading to severe respiratory and reproductive disease and causing substantial economic loss in animal husbandry (1). More human psittacosis cases with co-infection have been reported using next-generation sequencing (2). Clinically, *C. psittaci* infection presents symptoms similar to those of COVID-19, influenza, or human metapneumovirus, including high fever and pneumonia (3). Furthermore, *C. psittaci* infection suppresses host immunity and exacerbates the risk of secondary infection by impairing macrophage function and promoting the interleukin 10 (IL-10) expression, contributing to chlamydial infection and immune evasion (4, 5).

Like other intracellular pathogens such as *M. tuberculosis* (6), *Coxiella burnetii* and *Brucella abortus* (7), *C. psittaci* employs diverse cell receptors to invade the host (8). Several chlamydial outer-membrane components that interact with host cell receptors have been identified, including lipopolysaccharide (LPS) (9), the major outer membrane protein (MOMP) (10), and outer membrane complex protein B (OmcB) (11). Notably, the polymorphic membrane protein (Pmp) family has been demonstrated as adhesin that facilitates chlamydial infection (12). Compared to 1 *pmpG* gene of *Chlamydia trachomatis*, *C. psittaci* 6BC possesses 8 *pmpG* genes. Moreover, the identity of Pmp G remains highly conserved among different *C. trachomatis* serovars, while low similarity was detected in *C. psittaci* (12). This evidence suggests that *C. psittaci*-specific Pmp G might mediate multiple host tropisms during *C. psittaci* infection. In our prior investigation, *C. psittaci* Pmp17G and Pmp19G exhibited strong immunogenicity as potential vaccine candidate (13). As an adhesin, Pmp17G facilitates chlamydial invasion by binding the receptor of EGFR (14). However, the underlying function of Pmp19G remains poorly illustrated.

In our previous study, *C. psittaci* infection was determined to aggravate macrophage function by triggering Th2 polarization and contributing to secondary infections (4, 15). Therefore, the manipulation of macrophage function represents a critical step in *C. psittaci* infection. Within macrophage, autophagy serves as a crucial intracellular degradation process and a vital component of innate and adaptive immunity, combating intracellular pathogens and restricting bacterial spread (16). A few intracellular bacteria have successfully evolved various strategies to defend against autophagy and exploit this degradation process to acquire nutrition for their own development (17, 18). NOD-like receptors are crucial sensors for intracellular infections. The membrane association of NOD1 and NOD2 can recognize infection sites and initiate autophagic flow in macrophage (19). *Chlamydia pneumoniae* (*C. pneumoniae*) infection highly activated the NOD/Rip2 pathway and enhanced inflammatory response (20). Although the *nod1* gene itself is not directly associated with ischemic stroke, its polymorphism may serve as a potential marker for assessing stroke in the context of *C. pneumoniae*-positive serum (21). Additionally, wild-type and NOD1-knockout mice exhibit similar pathology following *C. trachomatis* or *C. muridarum* infection (22). However, NDO-like receptor as a receptor mediating early autophagy, its interaction with Pmp19G remains unknown. Regarding late autophagy, RAB7 is a small GTPase that governs the late stages of autophagy, including autophagosome maturation and fusion with lysosomes. Consequently, many pathogens target RAB7 to subvert host degradative pathways. One common strategy is to block RAB7 recruitment or activation on degradative membranes. SARS-CoV-2 accessory proteins ORF3a and ORF7a disrupt the homotypic fusion and vacuole protein sorting (HOPS) complex. This disruption impairs assembly of the RAB7–HOPS–SNARE fusion machinery, leading to accumulation of unfused autophagosomes and reduced autophagic flux (23, 24). Similarly, *Mycobacterium tuberculosis* interferes with the transition from Rab5 to Rab7 on pathogen-containing phagosomes by retaining early endosomal Rab GTPases and deploying effectors that prevent RAB7 enrichment. This arrest of phagosome maturation promotes intracellular persistence (25). Another strategy is post-translational reprogramming of RAB7. *Legionella pneumophila* injects Dot/Icm effectors that drive SUMOylation-dependent recruitment of RAB7 to the *Legionella*-containing vacuole, thereby rerouting late endosomal–lysosomal trafficking to establish a replication-permissive niche (26). These tactics converge on late autophagy to favor pathogen survival. However, the role of RAB7 in chlamydial infection is still obscure.

This study hypothesizes that *C. psittaci* exploits Pmp19G to regulate macrophage autophagy by binding NOD1 receptor and activating subsequent signaling pathway. By elucidating the mechanisms underlying host receptor manipulation and immune evasion, this research aims to provide novel insights into therapeutic strategies combating chlamydial infection.

## Materials and methods

### *C. psittaci* strain

*C. psittaci* 6BC strain (GenBank accession number CP002549) was donated from Professor Yimou Wu at the University of Southern China. The strain was propagated in Buffalo green monkey kidney cells at 37 °C with 5% CO_2_ for 72h. Live elementary bodies (EBs) were harvested and purified by density gradient centrifugation. The titer of inclusion-forming units (IFUs) was determined using the chlamydia direct immunofluorescence assay kit (IMAGEN™; Oxoid, Cambridge, UK). Finally, the live strain was stored in the sucrose phosphate glutamate (SPG) buffer at −80 °C (27).

### Expression and purification of recombinant Pmp19G protein

The *C. psittaci*-specific Pmp19G was cloned and expressed as previously described (28). In brief, *pmp19G* gene was amplified from *C. psittaci* 6BC genomic DNA and inserted into pET-28a plasmid (Novagen, Darmstadt, Germany), incorporating a His tag using Gibson assembly cloning kit (New England Biolabs, Massachusetts, USA). The plasmid was then transformed into *E. coli* DH5α (New England Biolabs, Massachusetts, US). Positive clones were selected and validated by PCR and double enzyme digestion. Plasmids were subsequently extracted using the Plasmid Mini Kit (Omega, Georgia, USA) according to the manufacturer’s instructions. Afterwards, the plasmids were transformed into *E. coli* Rossetta (DE3) (New England Biolabs, Massachusetts, USA) for expression. Finally, the recombinant protein was purified using Ni Sepharose High Performance (GE, Massachusetts, USA) and confirmed via SDS-PAGE and Western blot analysis. Endotoxin removal was performed using an affinity column (Thermo Fisher Scientific, Massachusetts, USA) and quantified residual LPS with a Limulus Amebocyte Lysate assay (Thermo Fisher Scientific, Massachusetts, USA). Endotoxin levels were consistently below 0.1 EU/mL, a threshold compatible with our cellular experiment.

### Cell preparation

For passage cells, the chicken macrophage HD11 cell line was donated by Prof. Qiao Jian’s lab at China Agricultural University and cultured in Dulbecco’s modified Eagle’s medium (DMEM) (Thermo Fisher Scientific, Massachusetts, USA) supplemented with 10% FBS, 1% sodium pyruvate, and 1% L-glutamine (Invitrogen, Massachusetts, USA) at 37 °C with 5% CO_2_ (29). For primary cells, alveolar macrophages were isolated from IL-10^-/-^ mice (Jackson Lab, Maine, USA), and wild-type C57BL/6 mice (Vitalstar Biotechnology Co., Ltd, Beijing, China) and NOD1^-/-^ mice, respectively. NOD1^-/-^ mice were generously provided by Associate Professor Zhihua Liu at Tsinghua University (30). All the mice were 6–8 weeks old and 18–20 g body weights. Mouse alveolar macrophages were harvested following previous description (31). Subsequently, cells were cultured in Roswell Park Memorial Institute (RPMI) 1640 medium (Thermo Fisher Scientific, Massachusetts, USA) supplemented with 10% FBS, 1% sodium pyruvate, and 1% L-glutamine (Invitrogen, Massachusetts, USA) and incubated at 37 °C with 5% CO_2_. 1 × 10^5^ cells per well were seeded in 24-well plates for the fluorescence-based autophagic flux assay. 5 × 10^5^ cells per well were seeded in 6-well plates for the Western blot, pull-down, and co-immunoprecipitation experiments.

### Fluorescence assay of autophagic flux

Autophagic flux was determined using the mRFP-GFP-LC3 adenovirus (Hanbio Co. Ltd., Beijing, China). Approximately 1×10^5^ macrophages were seeded in 24-well plates with cover glasses, and after overnight incubation, they were transfected with 5×10^5^ plaque-forming units (PFU) of adenovirus. The cells were cultured for 48h at 37 °C with 5% CO_2_ and then fixed with 4% paraformaldehyde for observation under a fluorescence microscope. The mRFP-GFP-LC3 adenovirus expresses monomeric red fluorescent protein (mRFP), and green fluorescent protein (GFP) were used to track LC3. During autophagosome formation, LC3 was observed as the dot stains in the cells, which were labeled with both mRFP and GFP, displaying yellow fluorescence due to overlapping stains. In the late stage of autophagy, autophagosomes were observed to fuse with lysosomes into autolysosomes by showing GFP quench in the acidic environment. Consequently, LC3 dots displayed only red fluorescence. Therefore, autophagic flux will be calculated by degerming yellow and red dots. The dot was obtained from 3 independent calculations at random for analysis.

### Cytokines detection

Approximately 1×10^5^ macrophages per well were infected with *C. psittaci* 6BC strain at a multiplicity of infection (MOI) of 1. Afterward, the cells were centrifuged at 700×*g* for 1h at 37 °C, followed by the addition of 1 μg/mL cycloheximide into each well. Cellular supernatants were collected at 6h and 12h post infection, respectively. Cytokines of IL-10, IL-4, and TNF-α were measured using commercial enzyme-linked immunosorbent assay (ELISA) kit following manufacturer’s instructions (Invitrogen, Massachusetts, USA).

### Western blot

After treatment with the *C. psittaci* 6BC strain or Pmp19G, macrophages were collected and lysed. The total protein loading amount of cell lysate is 20-30 μg. The samples were separated by 12% SDS-PAGE and transferred to a PVDF membrane (Thermo Fisher Scientific, Massachusetts, USA). After blocking with 5% bovine serum albumin at room temperature for 1h, the proteins were probed with primary antibodies including anti-His (1:5000), anti-LC3 (1:2000), anti-p62/SQSTM1 (1:2000), anti-LAMP (1:1000), anti-ATG16L1 (1:1000), anti-HA (1:200), anti-Nod1 (1:2000), anti-RAB7 (1:2000), anti-Tubulin (1:5000) or anti-GAPDH (1:5000) (Abcam, Cambridge, UK). Anti-IL-10R antibody (Affinity Biosciences, Cincinnati, USA) was validated species reactivity in chicken cells (**Supplementary Figure 2B**). Subsequently, goat anti-rabbit (1:10000) or goat anti-mouse (1:10000) secondary antibodies (Abcam, Cambridge, UK) were used. The bands were visualized using the Tanon Imaging system (Tanon Science & Tech, Beijing, China).

### Pull-down assay

Macrophages were treated with 5 μg/mL of recombinant His-Pmp19G at 37 °C for 6h. Nickel-nitrilotriacetic acid (Ni-NTA) beads were prewashed 3 times with 50 mM imidazole. The treated samples were then added to 200 μL of Ni-NTA beads and incubated overnight at 4 °C with agitation. Subsequently, the beads were washed 5 times with 70 mM imidazole. Finally, the proteins were eluted with 250 mM imidazole and then analyzed by Western blot. Samples without incubation with Ni-NTA beads were used as input controls.

### Expression of Pmp19G truncations and co-IP assay

To determine the interaction domain of Pmp19G, various Pmp19G truncations were constructed in plasmids, including pCMV-Pmp19G-N, pCMV-Pmp19G-C, and pCMV-Pmp19G. These plasmids were transfected into HD11 cells using Lipofectamine 3000 (Thermo Fisher Scientific, Massachusetts, USA). After 24h transfection, cells were washed three times with PBS and lysed in IP lysis buffer (Beyotime Biotech Ltd, Shanghai, China) at 4 °C for 30min. The cell lysates were then precleared with protein A/G PLUS agarose (Santa Cruz Biotechnology, Texas, USA) and incubated with anti-HA mAb and anti-NOD1 antibody at 4 °C for 8h, followed by three washes with IP buffer. Subsequently, Pmp19G truncations and NOD1 were identified by Western blot using HA-tagged or NOD1 antibodies.

### siRNA interference

NOD1 was knockdown by transfection of specific siRNA (GE, Massachusetts, USA). The transfection process was performed following manufacturer’s protocol. Briefly, 1×10^5^ HD11 cells per well were seeded into 24-well plates. The mixture of 25 nM siRNA, 1 μL Lipofectamine 3000, and 25 μL Opti-MEM™ Medium was added into cells. An equal amount of the mock siRNA was used as the negative control. After 48h incubation, cells were stimulated with 5 μg/mL of Pmp19G, and then samples were collected and analyzed at the corresponding time points as described above.

### Statistical analysis

Data were presented as means ± standard deviation (SDs). Statistical analysis and graphs were generated using GraphPad Prism 7 software (GraphPad Software Inc., California, USA) and Image J (National Institutes of Health, Maryland, USA). Student’s *t*-test was used to evaluate differences between two groups, while three or more groups were analyzed by one-way analysis of variance (ANOVA) with LSD *post-hoc* test. *P* values <0.05 were considered statistically significant.

## Results

### Expression and identification of *C. psittaci-*specific Pmp19G

**Figure 1:**
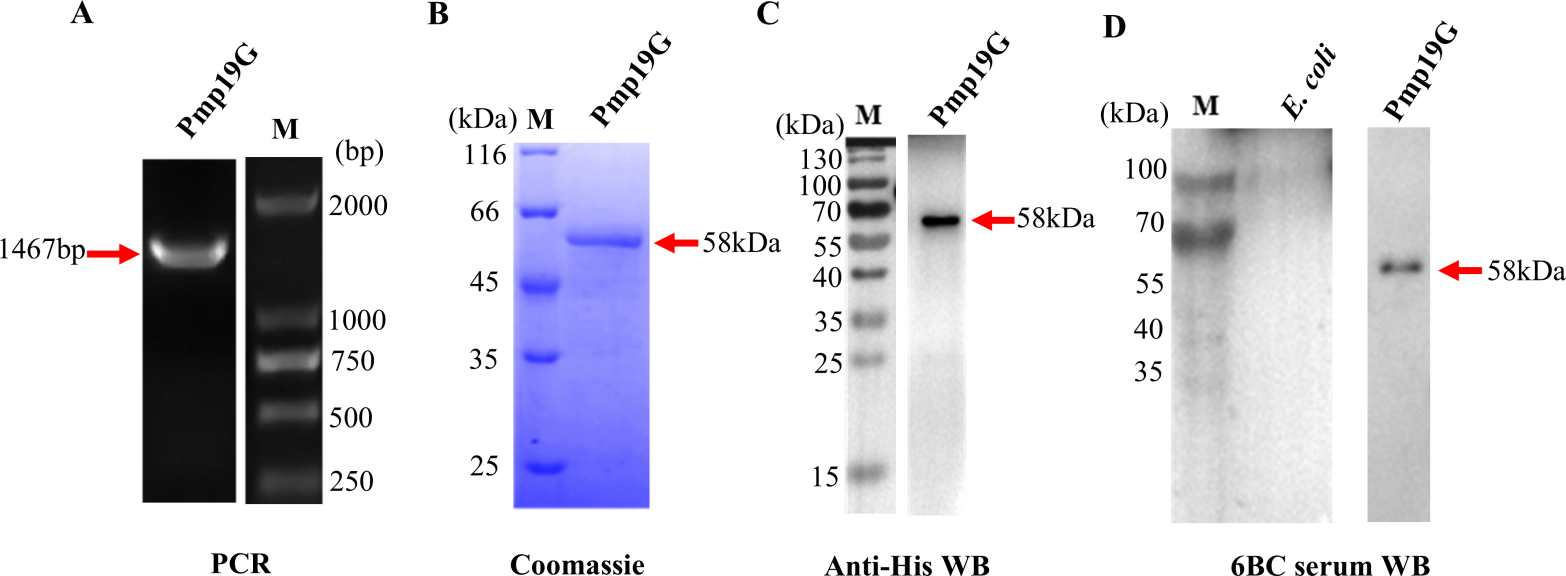
Expression and identification of *C*. *psittaci* Pmp19G. **(A)** The 1467 bp *pmp19G* gene fragment was amplified from *C*. *psittaci* 6BC genomic DNA by PCR. **(B)** Recombinant Pmp19G was expressed in *E. coli* Rossetta (DE3) and displayed a 58 kDa band by Coomassie blue staining. **(C)** The reaction of recombinant protein with anti-His antibody and positive *C*. *psittaci* serum was detected by Western blot. **(D)** No nonspecific cross-reaction to *E. coli* Rossetta (DE3) cells was observed.

Several *C. psittaci*-specific Pmp G family members have been successfully expressed and identified in our previous reports, including Pmp17G and Pmp20G (28, 32). To further investigate the role of Pmp 19G in regulating host receptor, we amplified the *pmp19G* gene from the *C. psittaci* 6BC strain and ligated it into the pET-28a vector (**Figure 1A**). The positive colonies were selected, and the target protein was expressed in the *E. coli* Rossetta (DE3) strain. After purification using a nickel column, recombinant Pmp19G produced 58 kDa band in SDS-PAGE stained with Coomassie brilliant blue (**Figure 1B**). Moreover, this protein was identified by Western blot using anti-His antibody and positive *C. psittaci* serum (**Figure 1C**). No cross-reaction with *E. coli* was observed (**Figure 1D**).

### *C. psittaci* infection promoted early autophagy in macrophages

**Figure 2:**
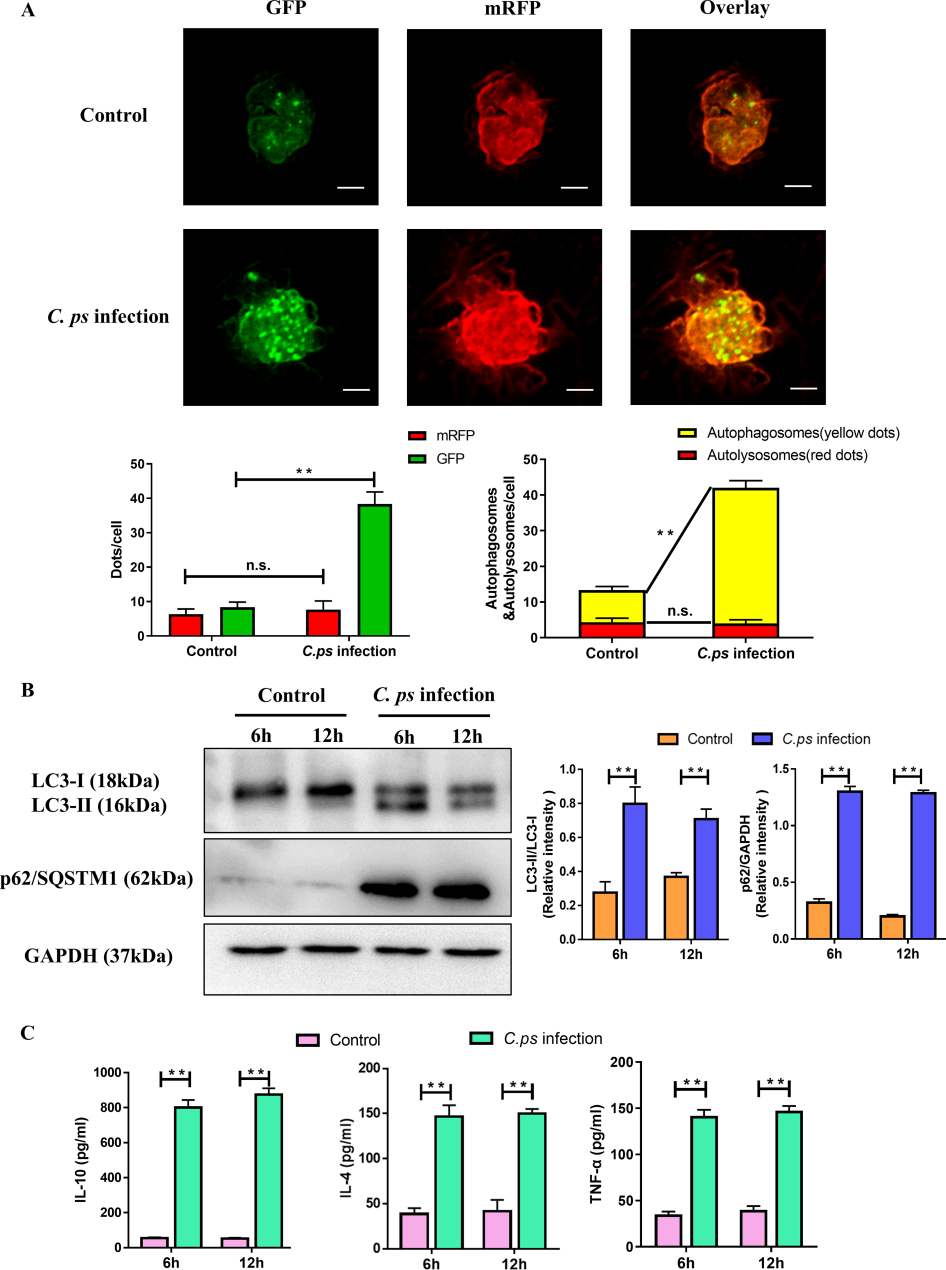
*C*. *psittaci* infection promoted early autophagy in macrophages. **(A)** HD11 cells were pretreated with mRFP-GFP-LC3 adenovirus and then infected with *C*. *psittaci* at MOI of 1. At 6 hpi, autophagic flow was determined by immunofluorescence. Yellow dots represent autophagosomes, red dots represent autolysosomes (Scale bar: 10µm). **(B)** LC3 I, LC3 II and p62/SQSTM1 were measured at 6 and 12 hpi by Western blot. Uninfected cells were served as the control group. GAPDH was used as the loading control. The intensity of the bands was quantified using ImageJ. Relative intensity was calculated as follows: relative intensity = indicated protein/GAPDH. **(C)** Secretion of IL-10, IL-4, and TNF-α were measured using ELISA kits at 6 and 12 hpi, respectively. Statistical analysis was performed by Student’s *t* test between the two groups, and the data from 3 independent experiments were expressed as the means ± SD (*C. ps*, *Chlamydia psittaci*; **P<0.01; n.s., no statistical significance).

At 6 hours post infection (hpi), higher number of green and yellow LC3 dots were observed in *C. psittaci* infection group compared to those of the control group, but no difference of the red LC3 dots was found, indicating that *C. psittaci* infection significantly enhanced the formation of autophagosomes instead of autolysosomes (**Figure 2A**). Additionally, the lysosomal associated membrane protein 1 (LAMP1) as a late autophagic protein has no difference compared to the control group during *C. psittaci* infection (**Supplementary Figure 1A**). Furthermore, *C. psittaci* infection promoted the conversion of LC3 I to LC3 II and the accumulation of p62/SQSTM1 compared to the control group at 6 hpi and 12 hpi, respectively (**Figure 2B**). Meanwhile, the productions of IL-10, IL-4, and TNF-α were significantly upregulated in the macrophages (**Figure 2C**). Therefore, *C. psittaci* infection induced early autophagy in the macrophages.

### Pmp19G is involved in the regulation of early autophagy in macrophages

**Figure 3:**
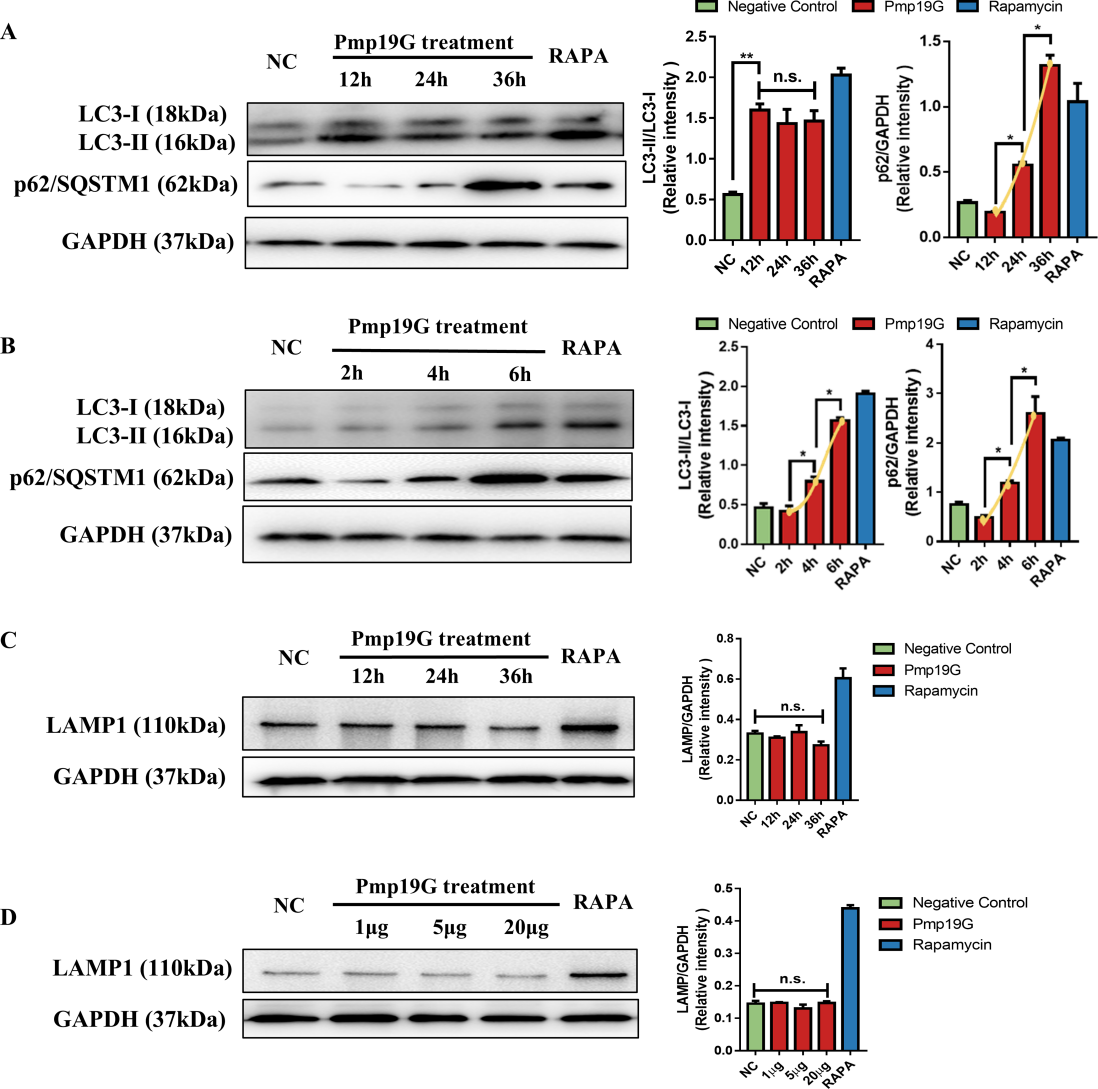
Pmp19G regulated early autophagy in macrophages. **(A)** HD11 cells were incubated with Pmp19G for 12, 24, and 36h, respectively. **(B)** HD11 cells were incubated with Pmp19G for 2, 4, and 6h, respectively. **(C)** HD11 cells were incubated with Pmp19G for 12, 24, and 36h, respectively. **(D)** HD11 cells were treated with 1 µg/mL, 5 µg/mL, and 20 µg/mL of Pmp19G. After samples collection, LC3 I, LC3 II, p62/SQSTM1 and LAMP1 were measured by Western blot. NC group as the negative control was no treatment, RAPA group as the positive control was treated with rapamycin. GAPDH was used as the loading control. Relative intensity was calculated as follows: relative intensity = indicated protein/GAPDH. Yellow curves were fitted to show the variation trend under each condition. Statistical analysis was performed by one-way ANOVA, and the data from 3 independent experiments were expressed as the means ± SD (NC, negative control; RAPA, rapamycin; *P<0.05, **P<0.01, n.s., no statistical significance).

To investigate the role of Pmp19G in the regulation of host autophagy, we performed long-term and short-term treatments. Although p62/SQSTM1 continuously increased, no significant difference was found in the conversion of LC3 I to LC3 II at 12h, 24h, or 36h, respectively (**Figure 3A**). Regarding short-term stimulation at 2h, 4h, and 6h, the conversion of LC3 I to LC3 II and p62/SQSTM1 increased significantly at different time points (**Figure 3B**). On the contrary, LAMP1 was not affected by serial doses of the Pmp19G treatment and different time points (**Figures 3C, D**). It suggests that Pmp19G regulated early autophagy in macrophage.

### Pmp19G was recognized by NOD1 receptor in macrophages

**Figure 4:**
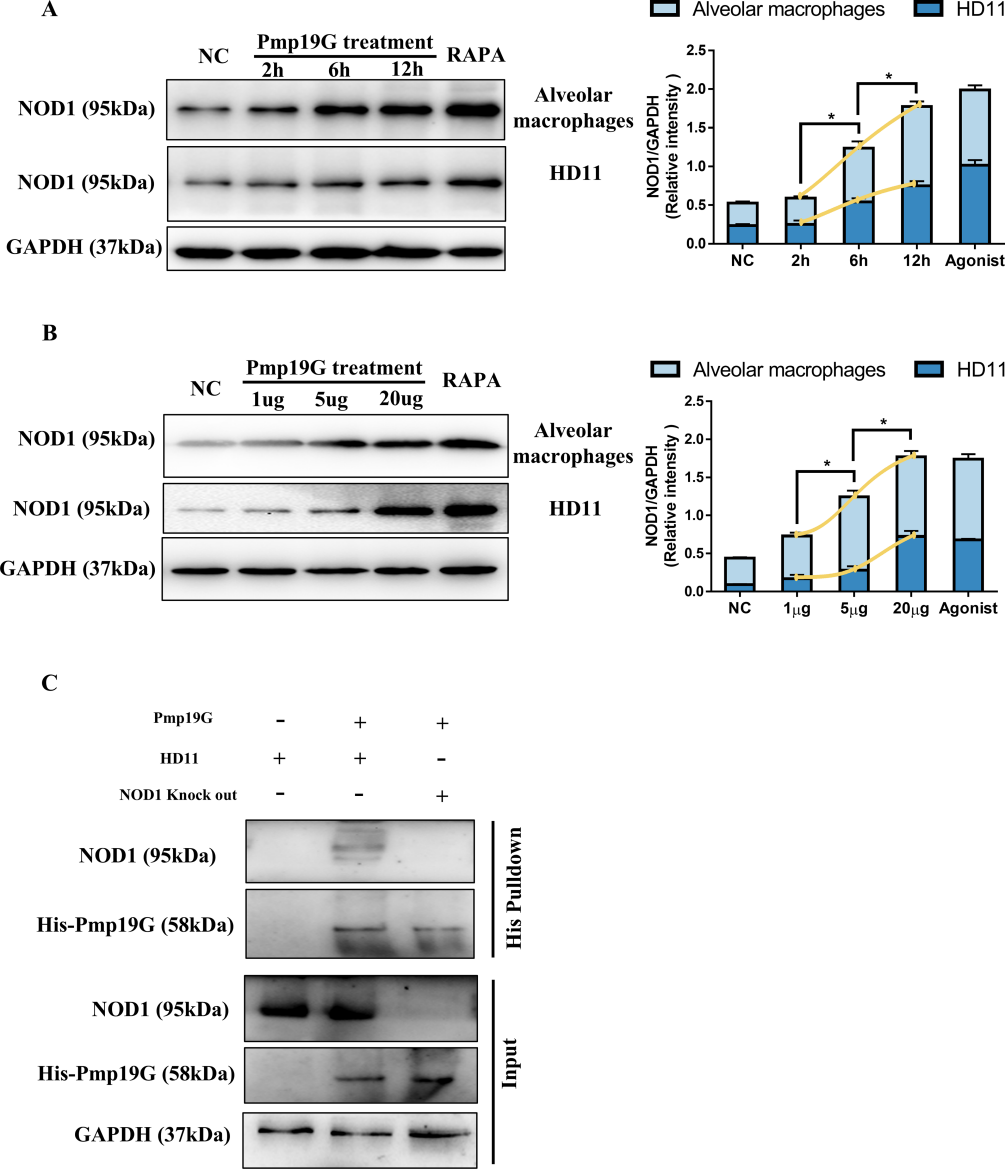
Pmp19G was recognized by NOD1 receptor in macrophages. **(A)** HD11 and primary alveolar macrophages were incubated with Pmp19G for 2, 6, and 12h, respectively. **(B)** HD11 and primary alveolar macrophage were treated with 1 µg/mL, 5 µg/mL, and 20 µg/mL of Pmp19G. After samples collection, the production of Nod1 was measured by Western blot. GAPDH was used as the loading control. Yellow curves were fitted to show the variation trend under each condition. **(C)** HD11 and NOD1^-/-^ cells were incubated with His-Pmp19G at 37 °C for 6h. Treated samples were added to 200 μL Ni-NTA beads and incubated overnight at 4 °C. Proteins were eluted with 250 mM imidazole and then analyzed by Western blot. The samples without incubation with Ni-NTA beads were used as input. Statistical analysis was performed by one-way ANOVA, and the data from 3 independent experiments were expressed as the means ± SD (NC, negative control; RAPA, rapamycin; *P<0.05).

We observed the interaction with NOD1 receptor both in passage and primary macrophages. After Pmp19G treatment, NOD1 expression was dramatically activated at 2h, 6h, and 12h, respectively (**Figure 4A**). A similar trend was observed post treatment with serial Pmp19G doses. Obviously, NOD1 expression gradually increased with the serial Pmp19G stimulations (**Figure 4B**). Furthermore, the interaction between NOD1 and Pmp19G was confirmed by pull-down assay. NOD1 band was recovered only when it was incubated with His-Pmp19G, and no nonspecific bands were observed in NOD1^-/-^ samples (**Figure 4C**). These findings indicated that Pmp19G could bind NOD1 receptor and induce potential functions.

### NOD1 receptor interacted with the N-terminal domain of Pmp19G

**Figure 5:**
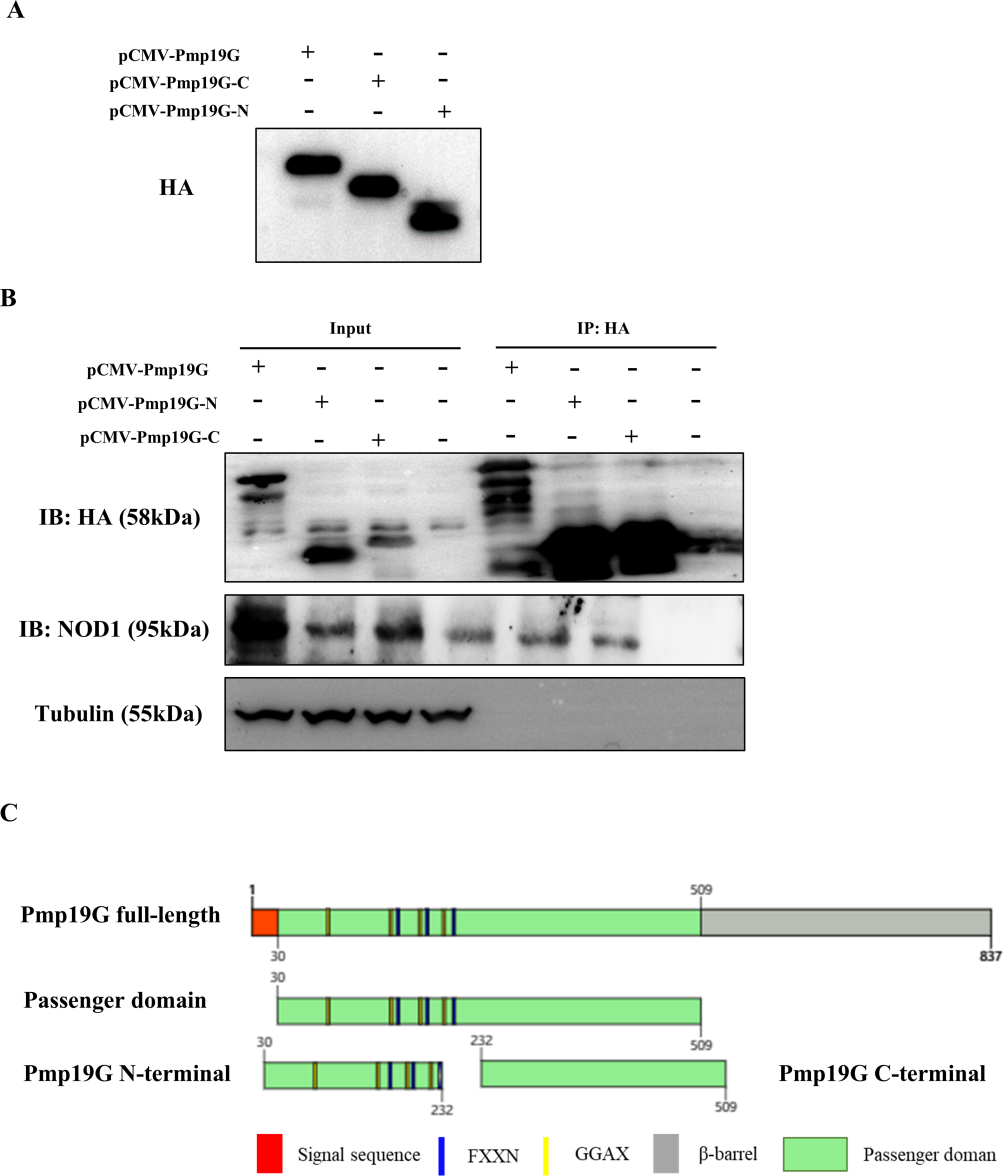
N-terminal domain of Pmp19G bound NOD1 receptor in macrophages. **(A)** Pmp19G N-terminal, C-terminal, and full-length were synthesized and constructed in plasmids, named pCMV-Pmp19G-N, pCMV-Pmp19G-C, and pCMV-Pmp19G. After 24h transfection, proteins were detected by Western blot using anti-HA antibody. **(B)** HD11 cells were transfected with pCMV-Pmp19G-N, pCMV-Pmp19G-C, and pCMV-Pmp19G, respectively. After 24h transfection, IP was performed with anti-HA antibody. Interacting proteins were determined by Western blot with the indicated antibody. An aliquot of each cell lysate was loaded as input to visualize the expression of the tagged proteins. **(C)** The schematic representation of Pmp19G was depicted with the signal sequence, passenger domain, and β-barrel domain. The truncated subdomains were Pmp19G N-terminal and C-terminal. Each of the tetrapeptide motifs GGAX (in yellow) and FXXN (in blue) were marked in the central passenger domain.

Firstly, different targeted truncations of Pmp19G were constructed and expressed using pCMV-HA vector. Subsequently, Pmp19G, Pmp19G N-terminal, and Pmp19G C-terminal were identified by Western blot using anti-HA antibody (**Figure 5A**). Co-immunoprecipitation further revealed that Pmp19G interacted with NOD1 receptor via its N-terminal in HD11(**Figure 5B**) and HEK293T cells (**Supplementary Figure 2A**). Based on the *pmp19G* gene sequence, we found three FxxN (where x is any amino acid) and four GGA(I/L/V) repeated tetrapeptide motifs in the N-terminal, but not in the C-terminal (**Figure 5C**). These results further implied that *C. psittaci* regulated host NOD1 receptor via Pmp19G N-terminal domain.

### NOD1 deficiency downregulated Pmp19G-induced early autophagy

**Figure 6:**
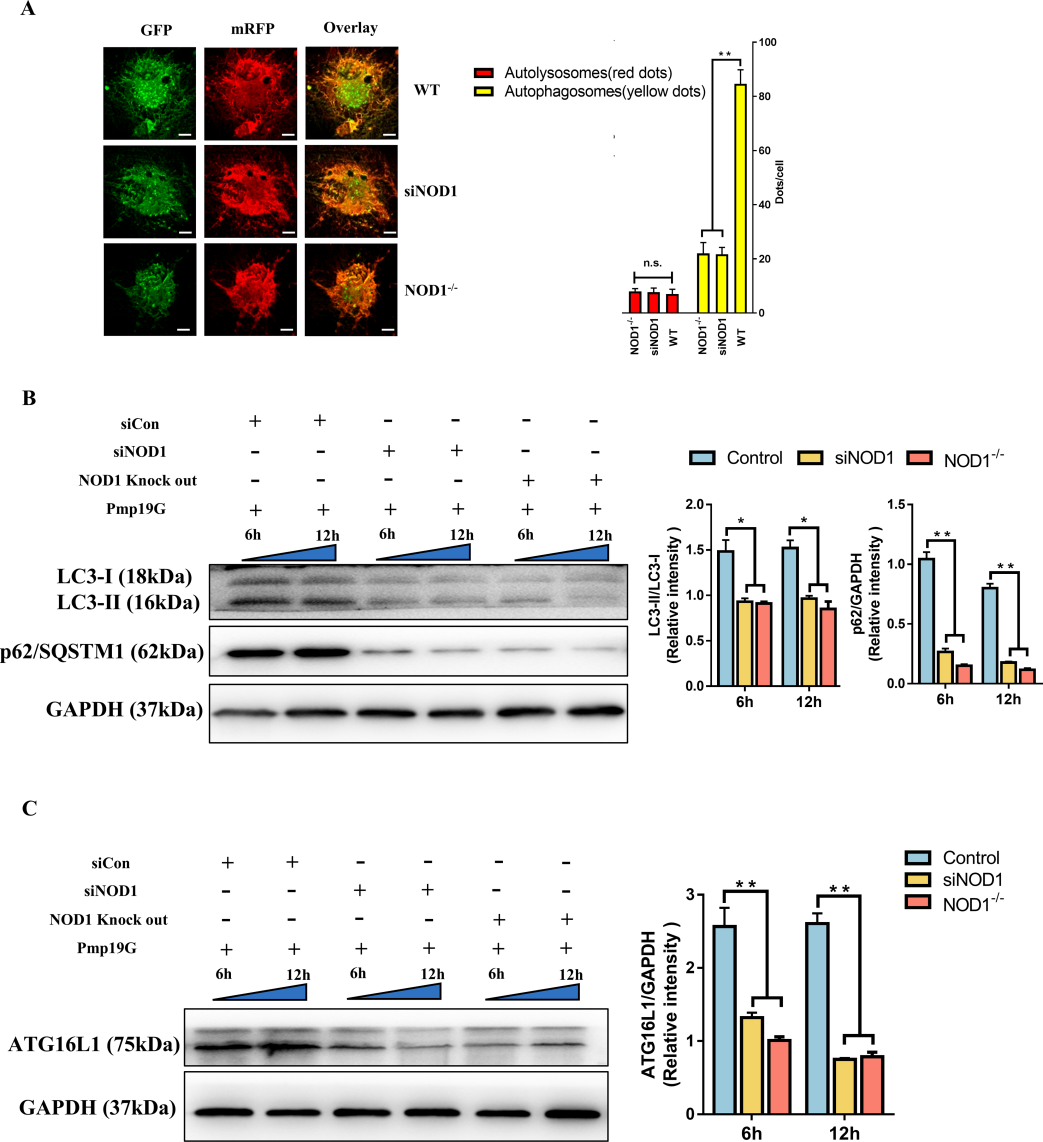
NOD1 deficiency impaired Pmp19G-mediated autophagy. **(A)** HD11 cells were pretreated with NOD1 siRNA, NOD1^-/-^ macrophages were isolated from the lung of NOD1 knock out mice. These passaged and primary cells were pretreated with mRFP-GFP-LC3 adenovirus and then incubated with Pmp19G. At 6h, the autophagic flow was determined by immunofluorescence. Yellow dots represent autophagosomes, and red dots represent autolysosomes (Scale bar: 10µm). **(B)** LC3 I, LC3 II and p62/SQSTM1 were measured at 6 and 12h by Western blot. **(C)** The production of ATG16L1 was measured at 6 and 12h by Western blot. GAPDH was used as the loading control. Statistical analysis was performed by one-way ANOVA, and the data from 3 independent experiments were expressed as the means ± SD (*P<0.05, **P<0.01, n.s., no statistical significance).

To further demonstrate the regulatory effect of Pmp19G on autophagy by binding the host NOD1 receptor, NOD1 knockdown (siNOD1) and NOD1 knockout (NOD1^-/-^) were constructed. Autophagosomes and autophagic flux were significantly downregulated in the NOD1^-/-^ and siNOD1 groups compared to the WT group (**Figure 6A**). Additionally, the conversion of LC3 I to LC3 II and the accumulation of p62/SQSTM1 were markedly decreased in the NOD1^-/-^ and siNOD1 groups at 6h and 12h, respectively (**Figure 6B**). ATG16L1 is a crucial autophagy related protein, can be recruited by NOD1 receptor to the membrane at the infection site. Interestingly, lower activation of ATG16L1 was observed in the NOD1^-/-^ and siNOD1 groups than in the control group (**Figure 6C**).

### IL-10 enhanced Pmp19G-activated autophagy via ATG16L1 signaling pathway

**Figure 7:**
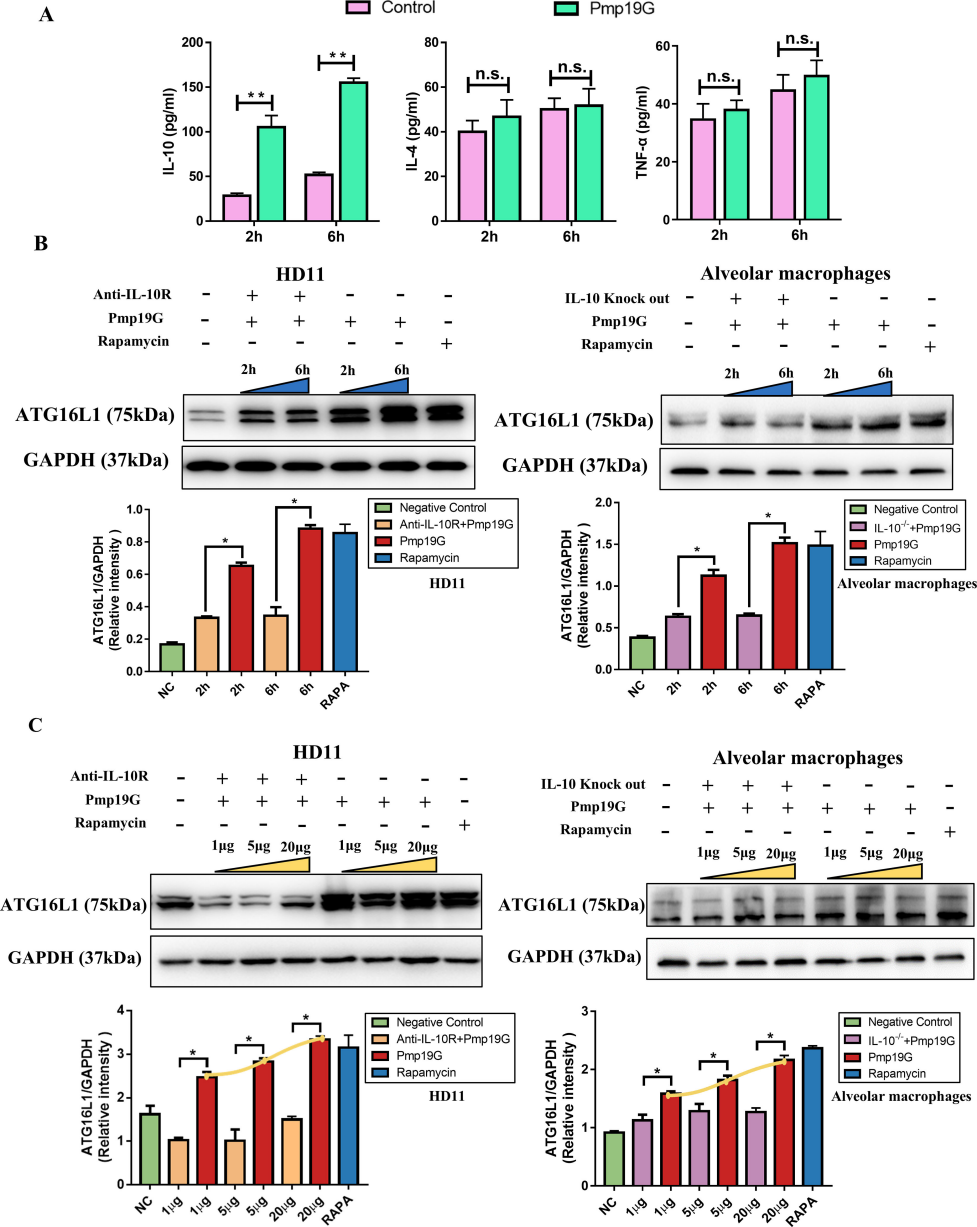
IL-10 enhanced Pmp19G-activated autophagy via ATG16L1 signaling pathway. **(A)** Secretion of IL-10, IL-4, and TNF-α were measured using ELISA kits at 2 and 6 hpi, respectively. **(B, C)** HD11 cells were pretreated with anti-IL-10R antibody to block IL-10, and alveolar macrophages were isolated from IL-10 knockout mouse. These passage and primary cells were incubated with Pmp19G for 2 and 6h, or treated with different concentration, including 1 µg/mL, 5 µg/mL, and 20 µg/mL. The production of ATG16L1 was measured by Western blot. GAPDH was used as the loading control. Yellow curves were fitted to show the variation trend under each condition. Statistical analysis was performed by Student’s *t* test between the two groups, and the data from 3 independent experiments were expressed as the means ± SD (*P<0.05, **P<0.01, n.s., no statistical significance).

In contrast with *C. psittaci* infection, Pmp19G stimulation only significantly enhanced the expression of IL-10 rather than IL-4 and TNF-α cytokines (**Figure 7A**). To understand the role of IL-10 in Pmp19G-activated autophagy, IL-10 receptor antibody and primary IL-10^-/-^ macrophages were used in the study. After Pmp19G treatment, ATG16L1 was significantly upregulated in the control group compared to the anti-IL-10R group and IL-10^-/-^ group at 2h and 6h, respectively both in HD11 and alveolar macrophages (**Figure 7B**). Moreover, ATG16L1 was markedly increased with dose-dependent effect. However, the anti-IL-10R group and IL-10^-/-^ group exhibited significant downregulation of ATG16L1 (**Figure 7C**). Therefore, Pmp19G induced early autophagy associated with the activation of ATG16L1 and IL-10.

### Pmp19G promoted the recruitment of RAB7 in late autophagy

**Figure 8:**
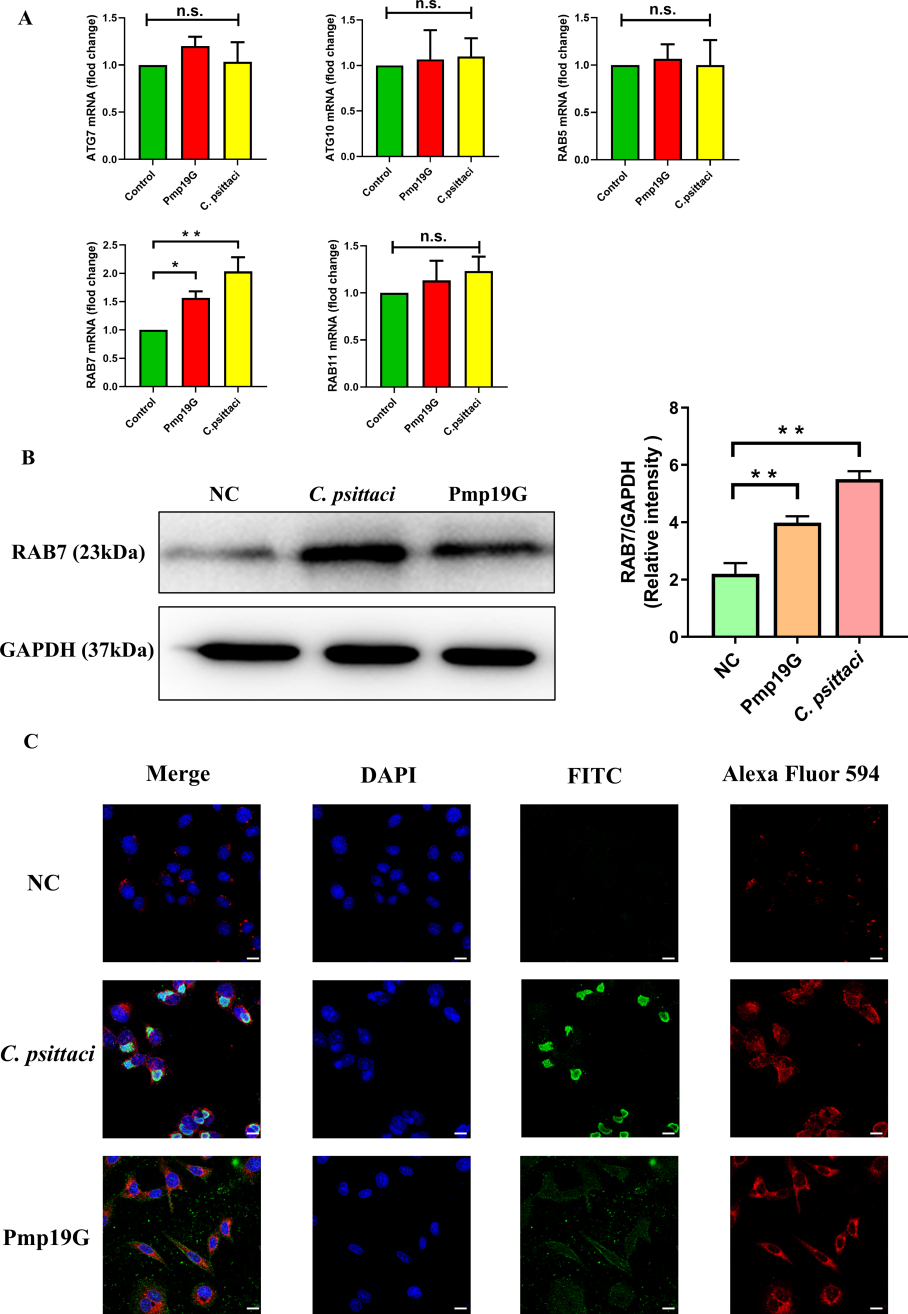
Pmp19G promoted the recruitment of RAB7 in late autophagy. **(A)** The transcription of *atg7*, *atg10*, *rab5*, *rab7*, and *rab11* genes were analyzed by qPCR following 6h post *C*. *psittaci* infection and Pmp19G stimulation. **(B)** The production of RAB7 was measured by Western blot. GAPDH was used as the loading control. **(C)** HD11 cells were fixed with methanol and incubated with antibodies. FITC represents anti-Pmp19G staining (green). Alexa Fluor 594 represents anti-RAB7 staining (red). DAPI represents nuclear staining (blue). Co-localizations were determined by multiplex immunofluorescence (Scale bar: 10 µm). Statistical analysis was performed by one-way ANOVA, and the data from 3 independent experiments were expressed as the means ± SD (*P<0.05, **P<0.01, n.s., no statistical significance).

To investigate the effect of Pmp19G on late autophagy of macrophages, we detected several late genes, including the family of the autophagy-related genes and Rab genes. Interestingly, *rab7* gene was significantly expressed during *C. psittaci* infection and Pmp19G stimulation (**Figure 8A**). Furthermore, RAB7 was activated with Pmp19G treatment and *C. psittaci* infection by Western blot assay (**Figure 8B**). Moreover, RAB7 was recruited around the *C. psittaci* and Pmp19G by co-location assay (**Figure 8C**). It suggested that Pmp19G promoted RAB7 to be hijacked for nutrient supply in the late autophagy.

## Discussion

**Figure 9:**
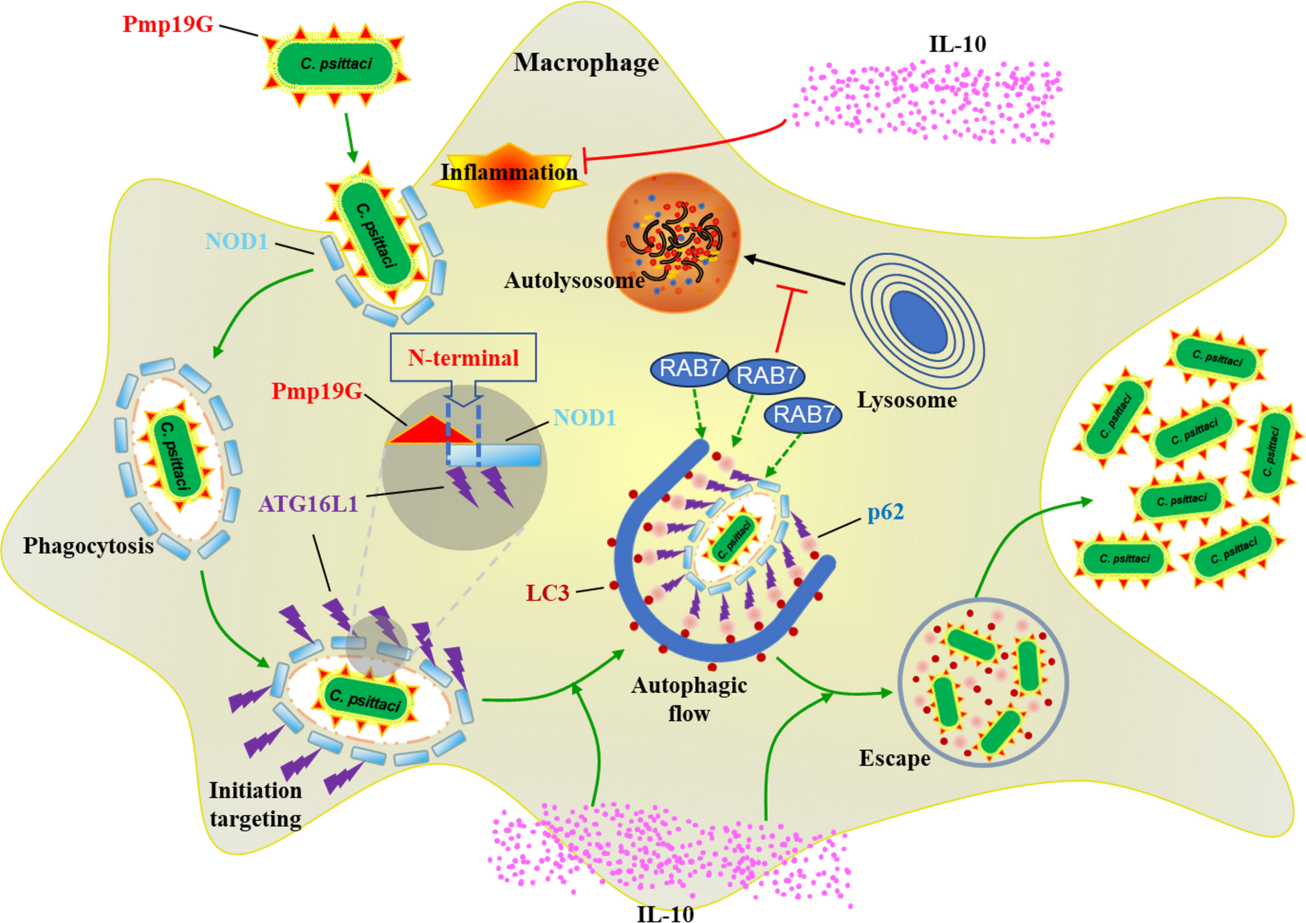
Schematic diagram. *C. psittaci* utilizes Pmp19G N-terminal to bind NOD1 receptor, mediating the early autophagy via NOD1-ATG16L1 and IL-10 signaling pathways. Moreover, IL-10 contributes to the activation of ATG16L1, promoting the autophagic flow. In late autophagy, Pmp19G promotes RAB7 to be hijacked in the late autophagy. This regulation mechanism could be associated with nutrient acquisition and immune evasion during *C. psittaci* infection. The green line denotes promotion, while the red line denotes suppression.

In this study, we investigated the regulation between *C. psittaci-*specific Pmp19G and host NOD1 receptor. Pmp19G N-terminal can interact with host NOD1 receptor in macrophage. Upon Pmp19G stimulation, NOD1-ATG16L1 and IL-10 signaling pathways were significantly activated and contributed to early autophagy in the macrophage. Pmp19G promoted autophagic flux, including a higher conversion of LC3 I to LC3 II and the accumulation of p62/SQSTM1. Furthermore, Pmp19G was found to promote the recruitment of RAB7 in late autophagy. Our findings provide new insights into the role of Pmp19G in mediating the early- and late- autophagy in macrophages by binding NOD1 receptor and manipulating ATG16L1 and RAB7 signaling pathways, contributing to a crucial role in *C. psittaci* invasion and persistent infection (**Figure 9**).

p62 accumulation is often interpreted as a block in autophagosome–lysosome fusion or degradation, our data indicate that *C. psittaci* Pmp19G primarily stimulates early autophagosome formation rather than impairing late-stage autolysosome degradation. Specifically, we observed increased LC3-II and GFP-LC3 puncta as early as 2h post-treatment, but lysosomal membrane marker LAMP1 were unaffected. It suggests that the late autophagic flux is blocked leading to accumulation of p62. In contrast, pharmacological blockade of autophagosome maturation (e.g., bafilomycin A1) sustained p62 accumulation and reduced LC3 turnover (33, 34). Thus, the early rise in p62 in our system reflects increased cargo loading into nascent autophagosomes, not defective degradation.

*C. psittaci* has a wide range of hosts, infecting birds, livestock and humans. Moreover, *C. psittaci* displays tropism for different tissues, leading to multiple lesions in severe patients (35). Among the identified *C. psittaci* genes, *pmpG* genes exhibit high mutation rates and cross-species characteristics. Single nucleotide polymorphism (SNP) plays a crucial role in chlamydial host adaptation, SNPs are mainly located in the *pmpG* gene cluster, suggesting the association of *pmpG* genes with host tropism (36). Adhesion is considered the initial step for triggering chlamydial invasion, and several chlamydial proteins have been identified as adhesins that attach to human cells and facilitate chlamydial entry. However, the specific mechanism of host-bacteria remains obscure. Our study revealed that *C. psittaci* Pmp19G mediated the interaction with host NOD1 receptor by pull-down and Co-IP assays. Furthermore, the N-terminal domain of Pmp19G was identified to bind NOD1 receptor instead of the C-terminal, suggesting its potential role in adhering and regulating host receptor during *C. psittaci* infection. Moreover, the infectivity was significantly reduced after *C. psittaci* was pre-treated with specific Pmp19G antibodies compared to that of the control group and MOMP group (**Supplementary Figure 3A**). It suggested that Pmp19G as a critical adhesins to promote initial *C. psittaci* infection.

Previous reports indicated that NOD-like receptors regulated immune responses (37). Peptidoglycan is commonly considered a specific ligand for NOD-like receptors. For instance, the Salmonella type III secretion system effector protein SipA can activate NF- κB via a NOD1 and NOD2-dependent signaling pathway (38), while SopE activates the Rho GTPases and then interacts with NOD1, leading to downstream signal activation (39). Given NOD1 being a cytosolic receptor, interaction between Pmp19G and NOD1 were identified in the study. Similarly, the secretion system ESX-1 of *M. tuberculosis* can cause damage to cell membrane, leading to the activation of NOD2 (40). Therefore, Pmp19G-NOD1 interaction might be crucial for regulating bacterial infection. This evidence suggests that Pmp19G could facilitate *C. psittaci* invasion via recognizing member receptors and achieve persistent infection in macrophage by binding NOD1 receptor.

Post *C. psittaci* invasion, multiple host cell surface and secreted components (Pmps, MOMP, LPS, etc.) are exposed that together engage several pattern-recognition receptors and elicit a mixed cytokine response (IL-10, IL-4, TNF-α). In contrast, purified Pmp19G is a single ligand that preferentially signals via a more limited receptor set, producing a more restricted cytokine profile such as IL-10 induction. IL-10 can also have feedback inhibitory effects on pro-inflammatory cytokines (e.g., TNF-α), further contributing to the differences. In our study, the activations of IL-10 and NOD1-ATG16L1 signaling pathways were observed after Pmp19G stimulation in a dose-dependent manner. ATG16L1 is well-known for its role in early autophagy, contributing to the formation of LC3-associated membranes (41). These findings align with our autophagic flow results, as we observed an increase in early autophagosome formation, but not in autolysosomes during the late stage. Moreover, LAMP1 as a biomarker of late autophagy was unaffected. These results suggest that ATG16L1 activation could facilitate *C. psittaci* replication after entry. Previous study has shown that *C. trachomatis* effector CT622 targeted ATG16L1 to disrupt cellular traffic for its own growth (42). Additionally, ATG16L1-deficient macrophages displayed increased uptake and elimination of uropathogenic *E. coli in vitro* (43). ATG16L1 mutant mice showed rapid bacterial clearance and epithelial recovery *in vivo* (44). These findings indicate that ATG16L1 potentially contributes to bacterial infection. On the other hand, ATG16L1 has also been demonstrated to negatively regulate NOD1 and NOD2-driven inflammation by blocking activation of Rip2 in response to invasive bacterial pathogens (19). More interestingly, the crucial inflammatory suppressor IL-10 was significantly upregulated in the study. In the previous report, IL-10^-/-^ promoted mice’s survival following *C. psittaci* infection by enhancing secretions of TNF-α, IL-1β and IFN-γ (45). Blocking IL-10 inhibited the production of ATG16L1, suggesting that ATG16L1 and IL-10 possibly have a synergistic action in suppressing the inflammatory response, thereby promoting chlamydial survival within macrophages.

Most intracellular nutrients primarily exist in the form of macromolecules, which chlamydia cannot directly access. Therefore, the participation of host degradative processes becomes a crucial step to acquire small molecule nutrients. Involvement in host autophagy is an ideal way to support chlamydial growth within infected cells. However, autophagy also serves as a vital defensive mechanism. Especially, Xenophagy as a kind of selective macroautophage that specifically combats intracellular pathogens, restricting their development and spread, such as *Salmonella* (46), *E. coli* (47) and others. Consequently, many bacteria have evolved various strategies to counter host autophagy. For instance, *M. tuberculosis* manipulates autophagic flux and inhibits autophagosome maturation and fusion with lysosome (48). Brucella requires the autophagosome-like vesicle and exploit autophagic flux to complete its intracellular cycle (49). *C. trachomatis* infection also induces autophagy, whereas the fusion of vacuole to lysosome is blocked (50). Additionally, *C. trachomatis* utilizes LC3 to promote infection in human epithelial cells (51). These findings explain our result that the recruitment of RAB7 in late autophagy during *C. psittaci* infection and Pmp19G treatment. It suggests that autophagic flow associated proteins play roles beyond autophagy and could be hijacked for chlamydial development.

This study elucidates the regulation by which *C. psittaci* Pmp19G activates the NOD1-ATG16L1 and IL-10 signaling pathways during early autophagy and recruits RAB7 in late autophagy. These findings uncover a novel regulatory mechanism that facilities *C. psittaci* infection and immune evasion. Insights into these pathways may inform the development of host-directed therapies to control chlamydial infections.

## Data Availability

The original contributions presented in the study are included in the article/**Supplementary Material**. Further inquiries can be directed to the corresponding author.

## Supplementary material

Supplementary Figure 1
*C. psittaci* infection induced early autophagy in macrophages. **(A)** HD11 cells were infected with *C. psittaci* at MOI of 1. LAMP1 was measured at 6 and 12 hpi by Western blot. Uninfected cells were served as the control group. GAPDH was used as the loading control. The intensity of the bands was quantified using ImageJ. Relative intensity was calculated as follows: relative intensity = indicated protein/GAPDH. **(B)** HD11 cells were pretreated with mRFP-GFP-LC3 adenovirus and then incubated with rapamycin (50 nM). At 24h, autophagic flow was determined by immunofluorescence. Green dots represent autophagosomes, red dots represent autolysosomes (Scale bar: 10 µm). Statistical analysis was performed by one-way ANOVA, data were expressed as the means ± SD (NC, negative control; n.s., no statistical significance).

Supplementary Figure 2 Interaction between Pmp19G and NOD1 in a mammalian cell line. **(A)** HEK293T cells were transfected with pCMV-Pmp19G-N. After 24h transfection, IP was performed with anti-HA antibody. Interacting proteins were determined by Western blot with the indicated antibody. An aliquot of each cell lysate was loaded as input to visualize the expression of the tagged proteins. **(B)** The reagent specificity of IL-10R was tested in chicken (HD11) and mammalian (HEK293T) cell lines by Western blot.

Supplementary Figure 3 Blocking Pmp19G reduced *C. psittaci* infectivity. **(A)** HD11 cells were pretreated with IgG, MOMP, and Pmp19G antibodies and then infected with *C. psittaci* at MOI of 1. At 48 hpi, the relative infection rate was determined by immunofluorescence. The red represents cells; the green represents chlamydia (Scale bar: 20 µm). Statistical analysis was performed by one-way ANOVA, and the data from 3 independent experiments were expressed as the means ± SD (**P*<0.05).

## References

[1] Beeckman DS Vanrompay DC. Zoonotic chlamydophila psittaci infections from a clinical perspective. Clin Microbiol Infect. (2009) 15:11–7. doi: 10.1111/j.1469-0691.2008.02669.x, PMID: 19220335

[2] Chen X Cao K Wei Y Qian Y Liang J Dong D. Metagenomic next-generation sequencing in the diagnosis of severe pneumonias caused by chlamydia psittaci. Infection. (2020) 48:535–42. doi: 10.1007/s15010-020-01429-0, PMID: 32314307 PMC7223968

[3] Yin Q Li Y Pan H Hui T Yu Z Wu H. Atypical pneumonia caused by chlamydia psittaci during the Covid-19 pandemic. Int J Infect Dis. (2022) 122:622–7. doi: 10.1016/j.ijid.2022.07.027, PMID: 35842216 PMC9276535

[4] Chu J Guo Y Xu G Zhang Q Zuo Z Li Q. Chlamydia psittaci triggers the invasion of H9n2 avian influenza virus by impairing the functions of chicken macrophages. Anim (Basel). (2020) 10:722. doi: 10.3390/ani10040722, PMID: 32326284 PMC7222846

[5] Li Q Li X Quan H Wang Y Qu G Shen Z. Il-10(-/-) enhances Dcs immunity against chlamydia psittaci infection via Ox40l/Nlrp3 and Ido/Treg pathways. Front Immunol. (2021) 12:645653. doi: 10.3389/fimmu.2021.645653, PMID: 34093535 PMC8176032

[6] Liang C Li X Li Q Zhang X Ren W Yao C. Clinical isolates of mycobacterium tuberculosis with different genotypes exhibit distinct host macrophage responses *in vitro* . J Med Microbiol. (2022) 71:1604. doi: 10.1099/jmm.0.001604, PMID: 36748527

[7] Czibener C Merwaiss F Guaimas F Del Giudice MG Serantes DAR Spera JM. Biga is a novel adhesin of brucella that mediates adhesion to epithelial cells. Cell Microbiol. (2016) 18:500–13. doi: 10.1111/cmi.12526, PMID: 26400021

[8] Henderson IR Lam AC. Polymorphic proteins of chlamydia spp. - autotransporters beyond the proteobacteria. Trends Microbiol. (2001) 9:573–8. doi: 10.1016/S0966-842x(01)02234-X, PMID: 11728862

[9] Ajonuma LC Fok KL Ho LS Chan PKS Chow PH Tsang LL. Cftr is required for cellular entry and internalization of chlamydia trachomatis. Cell Biol Int. (2010) 34:593–600. doi: 10.1042/Cbi20090227, PMID: 20178459

[10] Mehlitz A Rudel T. Modulation of host signaling and cellular responses by chlamydia. Cell Commun Signal. (2013) 11:90. doi: 10.1186/1478-811x-11-90, PMID: 24267514 PMC4222901

[11] Moelleken K Hegemann JH. The chlamydia outer membrane protein omcb is required for adhesion and exhibits biovar-specific differences in glycosaminoglycan binding. Mol Microbiol. (2008) 67:403–19. doi: 10.1111/j.1365-2958.2007.06050.x, PMID: 18086188 PMC2229832

[12] Favaroni A Trinks A Weber M Hegemann JH Schnee C. Pmp repertoires influence the different infectious potential of avian and mammalian chlamydia psittaci strains. Front Microbiol. (2021) 12:656209. doi: 10.3389/fmicb.2021.656209, PMID: 33854490 PMC8039305

[13] Li Q Chen S Yan Z Fang H Wang Z He C. A novel intranasal vaccine with Pmpgs + Momp induces robust protections both in respiratory tract and genital system posting chlamydia psittaci infection. Front Vet Sci. (2022) 9:855447. doi: 10.3389/fvets.2022.855447, PMID: 35529835 PMC9072866

[14] Li X Zuo Z Wang Y Hegemann JH He C. Polymorphic membrane protein 17g of chlamydia psittaci mediated the binding and invasion of bacteria to host cells by interacting and activating Egfr of the host. Front Immunol. (2021) 12:818487. doi: 10.3389/fimmu.2021.818487, PMID: 35173712 PMC8841347

[15] Chu J Li X Qu G Wang Y Li Q Guo Y. Correction: chlamydia psittaci pmpd-N modulated chicken macrophage function by triggering Th2 polarization and the Tlr2/Myd88/Nf-Kappab signaling pathway. Int J Mol Sci. (2020) 21:2639. doi: 10.3390/ijms21072639, PMID: 32290117 PMC7177311

[16] Clarke AJ Simon AK. Autophagy in the renewal, differentiation and homeostasis of immune cells. Nat Rev Immunol. (2019) 19:170–83. doi: 10.1038/s41577-018-0095-2, PMID: 30531943

[17] Keller MD Torres VJ Cadwell K. Autophagy and microbial pathogenesis. Cell Death Differ. (2020) 27:872–86. doi: 10.1038/s41418-019-0481-8, PMID: 31896796 PMC7205878

[18] Deretic V Levine B. Autophagy balances inflammation in innate immunity. Autophagy. (2018) 14:243–51. doi: 10.1080/15548627.2017.1402992, PMID: 29165043 PMC5902214

[19] Sorbara MT Ellison LK Ramjeet M Travassos LH Jones NL Girardin SE. The protein Atg16l1 suppresses inflammatory cytokines induced by the intracellular sensors Nod1 and Nod2 in an autophagy-independent manner. Immunity. (2013) 39:858–73. doi: 10.1016/j.immuni.2013.10.013, PMID: 24238340

[20] Shimada K Chen S Dempsey PW Sorrentino R Alsabeh R Slepenkin AV. The Nod/Rip2 pathway is essential for host defenses against chlamydophila pneumoniae lung infection. PLoS Pathog. (2009) 5:e1000379. doi: 10.1371/journal.ppat.1000379, PMID: 19360122 PMC2660273

[21] Tiszlavicz Z Somogyvari F Kocsis AK Szolnoki Z Sztriha LK Kis Z. Relevance of the genetic polymorphism of nod1 in chlamydia pneumoniae seropositive stroke patients. Eur J Neurol. (2009) 16:1224–9. doi: 10.1111/j.1468-1331.2009.02698.x, PMID: 19538217

[22] Welter-Stahl L Ojcius DM Viala J Girardin S Liu W Delarbre C. Stimulation of the cytosolic receptor for peptidoglycan, Nod1, by infection with chlamydia trachomatis or chlamydia muridarum. Cell Microbiol. (2006) 8:1047–57. doi: 10.1111/j.1462-5822.2006.00686.x, PMID: 16681844

[23] Miao G Zhao H Li Y Ji M Chen Y Shi Y. Orf3a of the Covid-19 virus Sars-Cov-2 blocks hops complex-mediated assembly of the snare complex required for autolysosome formation. Dev Cell. (2021) 56:427–42.e5. doi: 10.1016/j.devcel.2020.12.010, PMID: 33422265 PMC7832235

[24] Zhang Y Sun H Pei R Mao B Zhao Z Li H. The Sars-Cov-2 protein Orf3a inhibits fusion of autophagosomes with lysosomes. Cell Discov. (2021) 7:31. doi: 10.1038/s41421-021-00268-z, PMID: 33947832 PMC8096138

[25] Deretic V. Autophagy, an immunologic magic bullet: mycobacterium tuberculosis phagosome maturation block and how to bypass it. Future Microbiol. (2008) 3:517–24. doi: 10.2217/17460913.3.5.517, PMID: 18811236 PMC3225291

[26] Li C Fu J Shao S Luo ZQ. Legionella pneumophila exploits the endo-lysosomal network for phagosome biogenesis by co-opting sumoylated Rab7. PLoS Pathog. (2024) 20:e1011783. doi: 10.1371/journal.ppat.1011783, PMID: 38739652 PMC11115209

[27] Zuo ZH Zhang TY Guo YX Chu J Qu GG Miao LZ. Serosurvey of avian metapneumovirus, orithobacterium rhinotracheale, and chlamydia psittaci and their potential association with avian airsacculitis. Biomed Environ Sci. (2018) 31:403–+. doi: 10.3967/bes2018.053, PMID: 29866224

[28] Cui L Qu GG Chen Y Wu YX Wang CJ Cheng H. Polymorphic membrane protein 20g: A promising diagnostic biomarker for specific detection of chlamydia psittaci infection. Microbial Pathogenesis. (2021) 155:104882. doi: 10.1016/j.micpath.2021.104882, PMID: 33848596

[29] Chu J Li XH Qu GG Wang YH Li Q Guo YX. Chlamydia psittaci pmpd-N exacerbated chicken macrophage function by triggering Th2 polarization and the Tlr2/Myd88/Nf-Kappa B signaling pathway. Int J Mol Sci. (2020) 21:2003. doi: 10.3390/ijms21062003, PMID: 32183481 PMC7139469

[30] Yan RQ Liu ZH. Lrrk2 enhances Nod1/2-mediated inflammatory cytokine production by promoting rip2 phosphorylation. Protein Cell. (2017) 8:55–66. doi: 10.1007/s13238-016-0326-x, PMID: 27830463 PMC5233611

[31] Busch CJL Favret J Geirsdottir L Molawi K Sieweke MH. Isolation and long-term cultivation of mouse alveolar macrophages. Bio-Protocol. (2019) 9:e3302. doi: 10.21769/BioProtoc.3302, PMID: 31909091 PMC6944498

[32] Li Q Chen SY Yan ZQ Fang HX Wang ZX He C. A novel intranasal vaccine with pmpgs plus momp induces robust protections both in respiratory tract and genital system posting chlamydia psittaci infection. Front Veterinary Sci. (2022) 9:855447. doi: 10.3389/fvets.2022.855447, PMID: 35529835 PMC9072866

[33] Mizushima N Yoshimori T Levine B. Methods in mammalian autophagy research. Cell. (2010) 140:313–26. doi: 10.1016/j.cell.2010.01.028, PMID: 20144757 PMC2852113

[34] Klionsky DJ Abdel-Aziz AK Abdelfatah S Abdellatif M Abdoli A Abel S. Guidelines for the use and interpretation of assays for monitoring autophagy (4th edition)(1). Autophagy. (2021) 17:1–382. doi: 10.1080/15548627.2020.1797280, PMID: 33634751 PMC7996087

[35] Ravichandran K Anbazhagan S Karthik K Angappan M Dhayananth B. A Comprehensive review on avian chlamydiosis: A neglected zoonotic disease. Trop Anim Health Prod. (2021) 53:414. doi: 10.1007/s11250-021-02859-0, PMID: 34312716 PMC8313243

[36] Bachmann NL Fraser TA Bertelli C Jelocnik M Gillett A Funnell O. Comparative genomics of koala, cattle and sheep strains of chlamydia pecorum. BMC Genomics. (2014) 15:667. doi: 10.1186/1471-2164-15-667, PMID: 25106440 PMC4137089

[37] Woolls MK Mott MD Poole CS Gregory JA Ivester HM Allen IC. Innate immunity never “Nods” Off: Nlrs regulate the host anti-viral immune response. Immunol Rev. (2025) 330:e13429. doi: 10.1111/imr.13429, PMID: 39878363 PMC11776368

[38] Keestra AM Winter MG Klein-Douwel D Xavier MN Winter SE Kim A. A salmonella virulence factor activates the Nod1/Nod2 signaling pathway. Mbio. (2011) 2:e00266–11. doi: 10.1128/mBio.00266-11, PMID: 22186610 PMC3238304

[39] Hodge RG Ridley AJ. Regulating rho gtpases and their regulators. Nat Rev Mol Cell Bio. (2016) 17:496–510. doi: 10.1038/nrm.2016.67, PMID: 27301673

[40] Conrad WH Osman MM Shanahan JK Chu F Takaki KK Cameron J. Mycobacterial Esx-1 secretion system mediates host cell lysis through bacterium contact-dependent gross membrane disruptions. P Natl Acad Sci USA. (2017) 114:1371–6. doi: 10.1073/pnas.1620133114, PMID: 28119503 PMC5307465

[41] Wang C Bauckman KA Ross ASB Symington JW Ligon MM Scholtes G. A non-canonical autophagy-dependent role of the Atg16l1(T300a) variant in urothelial vesicular trafficking and uropathogenic Escherichia coli persistence. Autophagy. (2019) 15:527–42. doi: 10.1080/15548627.2018.1535290, PMID: 30335568 PMC6351132

[42] Hamaoui D Cosse MM Mohan J Lystad AH Wollert T Subtil A. The chlamydia effector Ct622/Taip targets a nonautophagy related function of Atg16l1. Proc Natl Acad Sci U.S.A. (2020) 117:26784–94. doi: 10.1073/pnas.2005389117, PMID: 33055216 PMC7604492

[43] Symington JW Wang C Twentyman J Owusu-Boaitey N Schwendener R Nunez G. Atg16l1 deficiency in macrophages drives clearance of uropathogenic E. Coli in an Il-1beta-dependent manner. Mucosal Immunol. (2015) 8:1388–99. doi: 10.1038/mi.2015.7, PMID: 25669147 PMC4532666

[44] Wang C Mendonsa GR Symington JW Zhang Q Cadwell K Virgin HW. Atg16l1 deficiency confers protection from uropathogenic Escherichia coli infection *in vivo* . Proc Natl Acad Sci U.S.A. (2012) 109:11008–13. doi: 10.1073/pnas.1203952109, PMID: 22715292 PMC3390880

[45] Li Q Li X Quan H Wang Y Qu G Shen Z. Il-10–/– enhances Dcs immunity against chlamydia psittaci infection via Ox40l/Nlrp3 and Ido/Treg pathways. Front Immunol. (2021) 12:645653. doi: 10.3389/fimmu.2021.645653, PMID: 34093535 PMC8176032

[46] Birmingham CL Smith AC Bakowski MA Yoshimori T Brumell JH. Autophagy controls salmonella infection in response to damage to the salmonella-containing vacuole. J Biol Chem. (2006) 281:11374–83. doi: 10.1074/jbc.M509157200, PMID: 16495224

[47] Brest P Lapaquette P Souidi M Lebrigand K Cesaro A Vouret-Craviari V. A synonymous variant in Irgm alters a binding site for Mir-196 and causes deregulation of Irgm-dependent xenophagy in Crohn’s disease. Nat Genet. (2011) 43:242–5. doi: 10.1038/ng.762, PMID: 21278745

[48] Chandra P Ghanwat S Matta SK Yadav SS Mehta M Siddiqui Z. Mycobacterium tuberculosis inhibits Rab7 recruitment to selectively modulate autophagy flux in macrophages. Sci Rep. (2015) 5:16320. doi: 10.1038/srep16320, PMID: 26541268 PMC4635374

[49] Starr T Child R Wehrly TD Hansen B Hwang S Lopez-Otin C. Selective subversion of autophagy complexes facilitates completion of the brucella intracellular cycle. Cell Host Microbe. (2012) 11:33–45. doi: 10.1016/j.chom.2011.12.002, PMID: 22264511 PMC3266535

[50] Yasir M Pachikara ND Bao X Pan Z Fan H. Regulation of chlamydial infection by host autophagy and vacuolar atpase-bearing organelles. Infect Immun. (2011) 79:4019–28. doi: 10.1128/IAI.05308-11, PMID: 21807906 PMC3187247

[51] Al-Younes HM Al-Zeer MA Khalil H Gussmann J Karlas A Machuy N. Autophagy-independent function of Map-Lc3 during intracellular propagation of chlamydia trachomatis. Autophagy. (2011) 7:814–28. doi: 10.4161/auto.7.8.15597, PMID: 21464618

