# Supplementary figures and images for: The N-terminal domain of Chlamydia psittaci Pmp19G modulates macrophage autophagy by targeting the NOD1 receptor and the ATG16L1–RAB7 signaling pathway
Source: Front Immunol. 2025 Sep 10;16:1645250. doi: 10.3389/fimmu.2025.1645250 (PMC12457404; doi:10.3389/fimmu.2025.1645250)

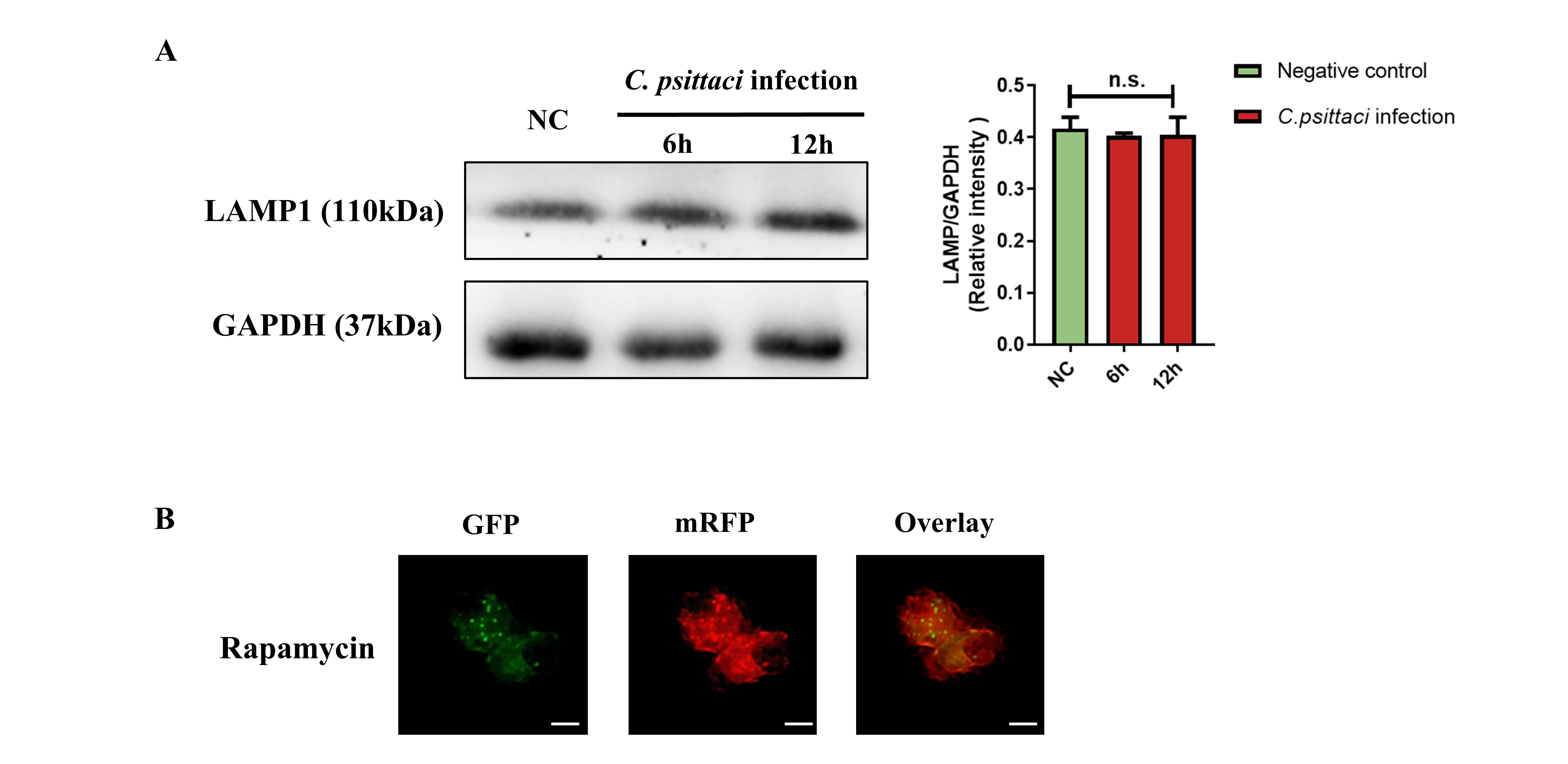

Supplement: Supplementary Figure 1 — C. psittaci infection induced early autophagy in macrophages. (A) HD11 cells were infected with C. psittaci at MOI of 1. LAMP1 was measured at 6 and 12 hpi by Western blot. Uninfected cells were served as the control group. GAPDH was used as the loading control. The intensity of the bands was quantified using ImageJ. Relative intensity was calculated as follows: relative intensity = indicated protein/GAPDH. (B) HD11 cells were pretreated with mRFP-GFP-LC3 adenovirus and then incubated with rapamycin (50 nM). At 24h, autophagic flow was determined by immunofluorescence. Green dots represent autophagosomes, red dots represent autolysosomes (Scale bar: 10 µm). Statistical analysis was performed by one-way ANOVA, data were expressed as the means ± SD (NC, negative control; n.s., no statistical significance). [file Image1.tif]

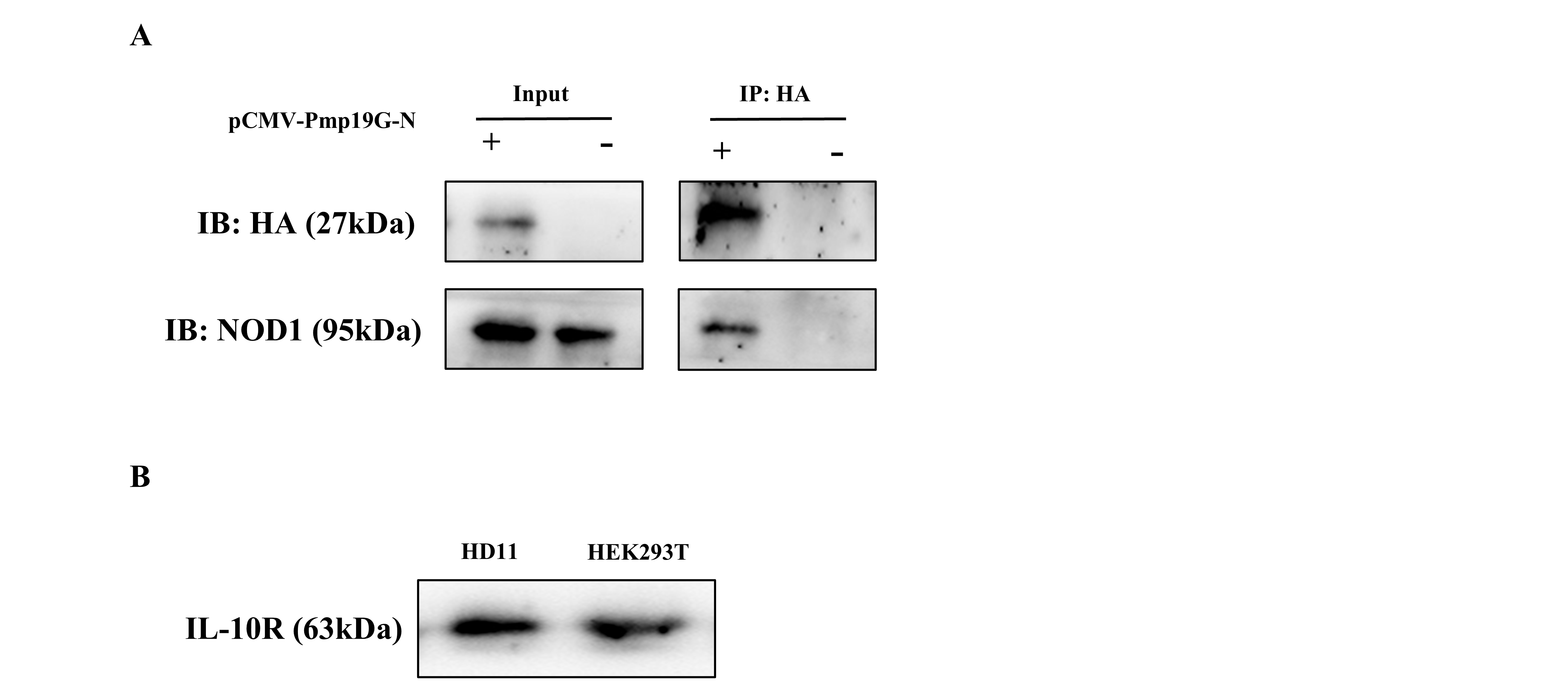

Supplement: Supplementary Figure 2 — Interaction between Pmp19G and NOD1 in a mammalian cell line. (A) HEK293T cells were transfected with pCMV-Pmp19G-N. After 24h transfection, IP was performed with anti-HA antibody. Interacting proteins were determined by Western blot with the indicated antibody. An aliquot of each cell lysate was loaded as input to visualize the expression of the tagged proteins. (B) The reagent specificity of IL-10R was tested in chicken (HD11) and mammalian (HEK293T) cell lines by Western blot. [file Image2.tif]

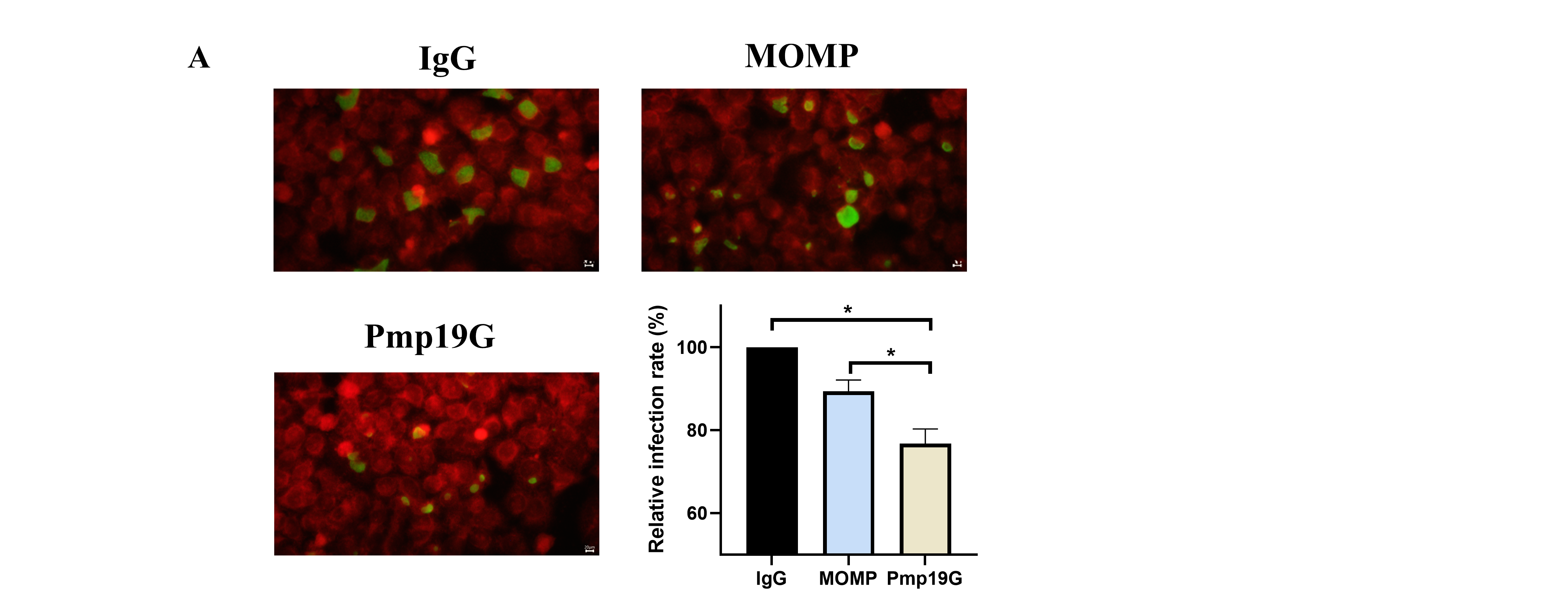

Supplement: Supplementary Figure 3 — Blocking Pmp19G reduced C. psittaci infectivity. (A) HD11 cells were pretreated with IgG, MOMP, and Pmp19G antibodies and then infected with C. psittaci at MOI of 1. At 48 hpi, the relative infection rate was determined by immunofluorescence. The red represents cells; the green represents chlamydia (Scale bar: 20 µm). Statistical analysis was performed by one-way ANOVA, and the data from 3 independent experiments were expressed as the means ± SD (*P<0.05). [file Image3.tif]
